# Use of e-cigarettes among public health students in Thailand: Embedded mixed-methods design

**DOI:** 10.18332/tid/152256

**Published:** 2022-09-07

**Authors:** Sarunya Benjakul, Saroj Nakju, Lakkhana Termsirikulchai

**Affiliations:** 1 Department of Health Education and Behavioral Sciences, Faculty of Public Health, Mahidol University, Bangkok, Thailand; 2 Faculty of Public Health, Ramkhamhaeng University, Bangkok, Thailand; 3 Tobacco Control Capacity Building Center, Thai Wellbeing Foundation, Bangkok, Thailand

**Keywords:** e-cigarettes, public health students, embedded mixed-method design

## Abstract

**INTRODUCTION:**

E-cigarettes are new tobacco products widely used among adolescents. Public health students are not only susceptible to e-cigarette use, but they should also serve as non-smoking role models. The study aimed to investigate the current situation of e-cigarette use and the factors associated with its use among public health students.

**METHODS:**

In this embedded mixed-methods design, the primary approach was a cross-sectional online survey. The samples were 2302 third-year public health students from 37 public health education institutes across Thailand. Stratified two-stage cluster random sampling was employed to select the subjects. Data were collected using self-administered questionnaires from January to March 2021. A checklist form was employed to collect qualitative data about teaching and learning activities related to tobacco control in June 2021. Descriptive statistics were used for data analysis, including inferential statistics regarding logistic regression.

**RESULTS:**

Overall, 3.9% (95% CI: 3.1–4.6) of the students currently used e-cigarettes in the past 30 days. The significant factors that could explain 43.4% of e-cigarette use were predisposing factors: being male (adjusted odds ratio, AOR=1.8; 95% CI: 1.0–3.3), having a neutral attitude toward e-cigarette use (AOR=2.2; 95% CI: 1.1–4.5), and not believing that public health professionals should serve as non-smoking role models for clients and the general public (AOR=2.3; 95% CI: 1.2–4.0). The enabling factor was having tried tobacco products (AOR=40.7; 95% CI: 19.1–87.1), and the reinforcing factor was having three or more close friends who smoke cigarettes (AOR=3.2; 95% CI: 1.8–5.8).

**CONCLUSIONS:**

Students’ behaviors should be modified through curriculum-based teaching and learning activities to develop negative attitudes toward e-cigarette smoking, increase students’ awareness as non-smoking role models, and establish smoke-free environments.

## INTRODUCTION

Although tobacco use is a significant health risk factor in populations globally, it constitutes a risk factor that can be prevented^1^. In Thailand, tobacco use is the first cause of premature death and disability in Thai populations^2^. Over the past 30 years (1991–2021), the smoking rate among Thais aged ≥15 years has declined, from 32.0% in 1991 to 17.4% in 2021^3^. However, to fulfill the global NCD targets, all nations must reduce tobacco use by 30% by 2025, compared with 2011^4^. That means Thailand’s smoking rate should reach 15.0% in 2025. As a result, in the next four years, starting in 2021, the smoking rate in Thailand should be reduced to 3.4% yearly. Therefore, preventing minors from becoming new smokers constitutes a crucial measure, as they represent a primary target population for the tobacco industry^5^. This measure corresponds to the WHO Framework Convention on Tobacco Control (WHO FCTC)^6^ and impacts the country’s capacity to produce quality human resources in the future.

Monitoring tobacco use is one of the measures recommended by the WHO FCTC. In 2005, the World Health Organization supported member countries in organizing the Global Health Professional Student Survey (GHPSS)^7^. Two phases of the survey were conducted in Thailand in 2006 and 2011^8^. Public health professions were among the health professions surveyed due to their essential roles in carrying out proactive health promotion and disease prevention programs and serving as effective role models for healthy lifestyles, including non-smoking behavior^9^.

Related surveys of the third-year public health students showed an increased smoking rate from 2.6% in 2006 to 4.3% in 2011. Additionally, in 2011, the smoking rate among public health students was the highest compared with other health professional students: medical doctors, nurses, pharmacists, dentists, medical technologists, and physiotherapists. In considering the teaching and learning of tobacco control content, a specific survey in 2006 showed that public health students received the least amount of teaching on tobacco control compared with other health professional students^8^. Presently, Thailand has enacted the Tobacco Products Control Act B.E.2560 (2017) to prevent minors from tobacco hazards^10^. However, this target group continues to be exposed to hazardous environments that push them to use tobacco products, especially new products such as electronic cigarettes (e-cigarettes) marketed to minors^11^. Those who can quickly assess this product through diverse types of online media^12,13^ can develop a positive attitude toward e-cigarettes and perceive that e-cigarettes produce less hazardous effects than traditional cigarettes^14^.

Therefore, in 2021, the Public Health Professional Alliance, part of the Thai Health Professional Alliance Against Tobacco (THPAAT), organized a smoking survey among public health students, focusing on e-cigarette use. The survey aimed to explore the current situation of e-cigarette use, teaching and learning about tobacco control, and the factors associated with e-cigarette use. The study’s findings will be beneficial in determining effective measures to prevent new smokers through teaching and learning activities in public health professional curricula. The practical outcomes of teaching and learning activities will focus on modifying public health students’ behavior from smoking initiation and quitting smoking. Because those students will become health professionals, their advanced knowledge and skills could contribute to sustainable tobacco control operations in the future.

## METHODS

### Research design, sample and procedure

An embedded mixed-methods study design^15^ was applied. A cross-sectional online survey was used as a primary dataset, and a qualitative approach using a checklist form generated the supplementary dataset.

The subjects comprised third-year public health students willing to provide online information. Students who did not attend the online class for any reason during the data collection period were not followed up and thus excluded. The termination criterion was applied to those students who initially consented to provide information but afterward felt uncomfortable doing so, either due to concerns about someone knowing their smoking behavior or concern about the confidentiality of the information provided, and withdrew from the research program.

The infinite population proportion formula in the sample size calculation application was applied to determine sample size^16^. The smoking rate in the past 30 days among the third-year public health students was 5.8%^17^. The error (d) was set at 0.01. The calculation yielded a sample size of 2099 people, which was then increased by 20%, resulting in a data collection sample size of 2520.

Thailand is typically divided into four geographical regions: central (including the Bangkok Metropolis), north, northeast, and south. A stratified two-stage cluster random sampling was employed as a sampling method. The overall 67 public health education institutes were divided into four strata, based on geographical location^18^. Simple random sampling was followed by drawing six to seven institutes in each geographical region, and 37 institutes were selected. In the second stage, the students who should be included in the survey were selected. Generally, a Bachelor’s degree in public health is a four-year program. The first two years are focused on basic sciences, with the remaining years devoted to public health core disciplines. Furthermore, the third year was the first year of field training, and various subjects could be integrated with tobacco control issues. As a result, the sampled students were all third-year students from each selected institute.

Regarding the supplementary dataset derived from the qualitative approach, 8 of 37 institutions, two from each region, were selected by simple random sampling. Then, 8 informants were chosen to provide information on teaching and learning activities arranged for public health students, either as administrators or representatives of instructors responsible for public health programs who were willing to provide information.

### Measurement

This study applied the PRECEDE-PROCEED Model’s phase 3 educational and ecological assessment^19^ as the research conceptual framework. The outcome variable measured was e-cigarette smoking, and the independent variables were predisposing, enabling, and reinforcing factors, as detailed below.

*Outcome variable*

The outcome variable was e-cigarette smoking in the past 30 days. This variable was divided into two groups: 1) currently not smoking e-cigarettes (0 days); and 2) currently smoking e-cigarettes (≥1 day).

*Predisposing factors*

These factors comprised demographic factors regarding age, geographical locations of the public health education institutions, beliefs that public health professionals should serve as non-smoking role models for clients and the general public (yes/no); and nine statements of attitude toward e-cigarette smoking such as ‘E-cigarette smoking shows modernity and being in the new generation’, ‘The modern image of e-cigarette arouses curiosity to try’, and ‘E-cigarettes are safer than traditional cigarettes’. A five-point rating scale was used for each statement, from 1=strongly disagree to 5=strongly agree. The summated score was grouped into three levels: negative attitude (<60%), moderate attitude (60–79%), and positive attitude (≥80%). The attitude scale was pretested with 30 public health students whose characteristics were similar to the sampled students. According to the scale reliability analysis, Cronbach’s coefficient alpha was equal to 0.898.

*Enabling factors*

In all, five statements aimed to assess enabling factors such as: ‘Ever tried using any tobacco products (never/ever)’; ‘Had attended learning and teaching activities on tobacco control, such as tobacco’s hazards, a technique of quitting cigarette smoking, marketing’s strategies of the tobacco industry (never/ever)’; ‘Had been exposed to tobacco control campaigns in an educational institute (never/ever)’; and ‘Had been informed about no smoking laws of every tobacco product including e-cigarette (no/yes)’. The respondents were asked to answer one choice for each statement.

*Reinforcing factors*

This part focused on questions regarding the number of close friends who smoked tobacco products. Three groups were developed according to the number of close friends mentioned: 0, 1–2, and ≥3.

Qualitative data were gathered after completing an online survey using a checklist form to indicate tobacco control activities organized with some explanation and the attached documents or pictures. The data were used to comprehend better the tobacco control teaching and learning activities included in the institute’s curricula. Five questions concerned two categories. The first category included learning design regarding the complete subjects containing content on tobacco control, the topics containing content on tobacco control in any subject, and student assignments. The second category included extracurricular activities concerning tobacco control campaigns organized in the faculty/department and community by the faculty/department.

### Ethics approval

This research has been approved by Srinakharinwirot University. The consent form was cited on the front page of the online survey. The students who clicked the ‘I accept’ button were then given access to the survey questions. When students clicked the ‘I refuse’ button, the form was skipped to the end and submitted. According to the ethics committee’s approval, a small token of appreciation, a coffee gift card, was given to students who completed the survey questions by drawing randomly from a computer system.

### Data collection

The following activities used the online self-administered questionnaires (Supplementary file) to collect data. First, the researcher clarified the aim and procedures for collecting data with the institutes’ representatives and determined the day and time of online teaching used to request the selected students complete the questionnaires. The URL and QR code of the questionnaire were then distributed to the institutes’ representatives. This activity took place in January 2021. Second, the institutes’ representatives explained the research objectives, procedures for answering questionnaire items and displayed the URL and QR code to students using two channels, the computer monitor of instructors’ online teaching and the LINE chat application. The selected students took 10–15 minutes to complete and submit their questionnaires. This activity occurred from February to March 2021. Finally, the research team received and verified the data through the computer system in March 2021.

After completing the quantitative data collection process, the qualitative data were gathered in June 2021 using the checklist form by sending an official letter to the selected administrators, asking them to contribute data by filling in the checklist forms, including the QR code. The research team retrieved and analyzed the data to support the quantitative data on teaching and learning activities about tobacco control.

### Statistical analysis

The Statistical Package for the Social Sciences (IBM SPSS Statistics, Version 18) was used to analyze the data. Descriptive statistics were used to describe the study variables. Inferential statistics, one-sample t-test, were also applied to determine the e-cigarette smoking rate and the 95% confidence interval (CI). Odds ratio (OR) with 95% CI by simple logistic regression and adjusted odds ratio (AOR) with 95% CI by multiple logistic regression were used to assess the association size between factors in the PRECEDE – PROCEED MODEL and e-cigarette smoking. Two-sided p<0.05 were used to determine statistical significance.

## RESULTS

The final sample comprised 37 public health education institutes (institute response rate: 100%). The total number of completed online questionnaires was 2302, more than the calculated minimum sample size of 2099. The research results were presented in four parts: demographic characteristics, e-cigarette smoking rate and behavior, learning and teaching about tobacco control, and the factors associated with e-cigarette smoking. The details are presented below.

### Demographic characteristics

Most subjects were females (90.4%), aged 19–21 years (83.0%), with a mean age of 21.1±0.7 years, 96.2% studied at government institutes, and 41.6% studied at institutes in northeast Thailand.

### E-cigarette smoking rate and behavior

In the past 30 days, 3.9% (95% CI: 3.1–4.6) of the subjects currently used an e-cigarette, with males using them four times more than females (12.3%; 95% CI: 7.9–16.6 vs 3.0%, 95% CI: 2.3–3.7). Similarly, the current e-cigarette smoking rate of those who had tried any tobacco products in their lifetime was significantly higher than that of those who never tried to smoke (20.8%; 95% CI: 16.7–24.8 vs 0.4%, 95% CI: 0.1–0.7) (Table 1).

**Table 1:** Prevalence of current e-cigarette use with 95% CI among the third-year public health students by selected demographic characteristics (N=2302)

*Characteristics*	*Prevalence of current e-cigarette use*	
	*%*	*95% CI*
**Total**	3.9	3.1–4.6
**Sex**		
Female	3.0	2.3–3.7
Male	12.3	7.9–16.6
**Age** (years)		
19–21	3.6	2.8–4.5
≥22	5.1	2.9–7.3
**Region**		
North	4.0	2.2–5.8
Northeast	4.1	2.8–5.3
Central (including Bangkok Metropolis)	4.3	2.6–5.9
South	2.3	0.6–3.9
**Had tried any smoked tobacco products**		
No	0.4	0.1–0.7
Yes	20.8	16.7–24.8

Of current e-cigarette users, 78.7% indicated that they only used e-cigarettes, 20.2% as dual users using both e-cigarettes and cigarettes, and 1.1% used all kinds of smoked tobacco products, e-cigarettes, cigarettes, and others such as baraku or shisha. Furthermore, 15.7% used e-cigarettes daily, an average of six times per day and each time with an average of 4.1 times inhaling. Regarding where they could buy e-cigarettes, 57.1% bought online, followed by 23.8% who mentioned places like sales agencies, asking friends to buy, or buying from friends.

### Learning and teaching about tobacco control

During their study at public health education institutes, 71.9% said they studied tobacco control. Almost all in this group (97.3%) had been taught about the hazardous effects of tobacco products. In comparison, 63.5% and 51.1% had been taught about quitting smoking and the marketing strategies of tobacco industries, respectively.

According to the qualitative data, eight sample institutes had similar approaches to teaching and learning about tobacco control. No core subject covered tobacco control in its entirety. Only topics or case studies were integrated into the institutes’ curricula core subjects. For example, the topics included health determinants, concepts, principles of promoting health under the Ottawa Charter of Health Promotion, strategies and methods of health education, and tobacco control laws. Furthermore, the number of sessions or time allotments (hour/time) to the contents incorporated in the core subjects were unidentified. In terms of field training programs, whether the public health programs organized by students in the community were relevant to tobacco control depended on the findings of community diagnosis. Regarding extracurricular activities, most programs organized anti-smoking campaigns associated with special events such as World No Tobacco Day, World Environment Day, and International Day Against Drug Abuse and Illicit Trafficking.

### Factors associated with e-cigarette smoking

The analysis using simple logistic regression showed that the following seven factors were significantly associated with third-year public health students’ e-cigarette use. The predisposing factors included being male (OR=4.6; 95% CI: 2.8–7.3, p<0.001), having a neutral attitude toward e-cigarette use (OR=4.7; 95% CI: 2.8–8.2, p<0.001), and not believing that public health professionals should serve as non-smoking role models for clients and the general public (OR=2.1; 95% CI: 1.2–3.5, p=007). The enabling factors were that they had tried to use any tobacco products in their lifetime (OR=62.4; 95% CI: 29.9–130.3, p<0.001) and had been exposed to anti-smoking campaigns in their public health education institutes (OR=0.5; 95% CI: 0.3–0.9, p=0.009). For the reinforcing factor, the number of close friends who smoked any tobacco products, 1–2 and ≥3, was found to provide more opportunities to use e-cigarettes than not having close friends who smoked any tobacco products (OR=3.2; 95% CI: 1.7–5.7, p<0.001 vs OR=13.2; 95% CI: 7.9–22.0, p<0.001, respectively).

According to the multiple logistic regression analysis, the Hosmer-Lemeshow test proved appropriate in explaining the variance (χ^2^=8.559, p=0.128). Five factors were significantly associated with e-cigarette smoking by 43.4%, namely, predisposing factors: being male (adjusted odds ratio, AOR=1.8; 95% CI: 1.0–3.3, p=0.038), having a neutral attitude toward e-cigarette use (AOR=2.2; 95% CI: 1.1–4.5, p=0.028) and not believing that public health professionals should serve as non-smoking role models for clients and the general public (AOR=2.3; 95% CI: 1.2–4.5, p=0.011). The enabling factor was those who had tried e-cigarettes in their lifetime (AOR=40.7; 95% CI: 19.0–87.1, p<0.001); and one reinforcing factor constituted those having ≥3 close friends who smoked any tobacco products (AOR=3.2; 95% CI: 1.8–5.8, p<0.001) (Table 2).

**Table 2:** Factors associated with current e-cigarette use among third-year public health students in 2021 (N=2302)

*Factors*	*OR (95% CI)*	*p*	*AOR (95% CI)*	*p*
***Predisposing factors***				
**Sex**				
Female (Ref.)	1		1	
Male	4.6 (2.8–7.3)	<0.001	1.8 (1.0–3.3)	0.038
**Age** (years)				
19–21 (Ref.)	1			
≥22	1.4 (0.9–2.4)	0.162		
**Region**				
North (Ref.)	1			
Northeast	1.0 (0.6–1.8)	0.958		
Central (Including Bangkok Metropolis)	1.1 (0.6–1.9)	0.848		
South	0.6 (0.2–1.3)	0.190		
**Attitude towards e-cigarette use**				
Negative (Ref.)	1		1	
Neutral	4.8 (2.8–8.2)	<0.001	2.2 (1.1–4.5)	0.028
Positive	1.5 (0.2–11.4)	0.693		
**Belief that public health professionals should be non-smoking role models**				
Yes (Ref.)	1		1	
No	2.1 (1.2–3.5)	0.007	2.3 (1.2–4.5)	0.011
***Enabling factors***				
**Had tried any tobacco product**				
No (Ref.)	1		1	
Yes	62.4 (29.9–130.3)	<0.001	40.7 (19.1–87.1)	<0.001
**Had attended a class on a topic related to tobacco control**				
Yes (Ref.)	1			
No	0.9 (0.5–1.4)	0.622		
**Had been exposed to anti-smoking campaigns in their public health education institutes**				
Yes (Ref.)	1		1	
No	0.5 (0.3–0.9)	0.009	0.6 (0.4–1.1)	0.079
**Had taken part in an anti-smoking campaign at the institute**				
Yes (Ref.)	1			
No	1.0 (0.6–1.7)	0.970		
**Perceived smoking any tobacco products including e-cigarettes in educational institutes is against the law**				
Yes (Ref.)	1			
No	0.3 (0.1–1.4)	0.124		
***Reinforcing factors***				
**Number of close friends who smoked**				
None (Ref.)	1		1	
1–2	3.2 (1.7–5.7)	<0.001	1.6 (0.8–2.9)	0.176
≥3	13.2 (7.9–22.0)	<0.001	3.2 (1.8–5.8)	<0.001

AOR: adjusted odds ratio.

## DISCUSSION

Currently e-cigarette users totaled 3.9% (95% CI: 3.1–4.6). Those who had tried smoked tobacco products in their lifetime were more likely to use e-cigarettes than those who had never tried smoking. Online stores were the main access point for obtaining e-cigarettes. This finding was similar to a study conducted among nursing students in northeast Italy (2.1%, 95% CI: 1.5–3.0)^20^. However, compared with related studies conducted in Thailand and other countries, the current use of e-cigarettes was fairly low. For example, e-cigarettes were used by 20.0% of all levels of undergraduate students in a health science faculty at one private university in Thailand^21^ and 20.6% of health professional students in one state in the US^22^. These findings are due to the high prevalence of e-cigarettes among adolescents in the US^23,24^.

Furthermore, in locations where social media via the internet are easily accessible, such as urban and economic areas, a high prevalence of e-cigarette use has been observed. Also, more availability for sales promotion advertising can be found at these sites^25^. Compared with traditional cigarettes, the taste and odor of e-cigarettes could generate a significant difference^26,27^. Regarding the source for obtaining e-cigarettes, Thailand has had a law prohibiting the import and sale of e-cigarettes since 2014^28^, and the Tobacco Control Act of 2017 comprehensively bans advertising and promotion of e-cigarettes^10^. However, laws not permitting online commerce can always be found. According to a national smoking behavior survey conducted by the National Statistical Office in 2021, 59.9% of current e-cigarette users aged 15–24 years purchased e-cigarettes online^3^. This issue could result from a staff shortage and improper law enforcement concerning online selling system technology.

Regarding learning-teaching about tobacco control, the qualitative data could be used to support the study’s findings. Topics on tobacco control were only integrated into the core subjects, whereas the number of sessions and time allotment could not be identified. In addition, no continuous extracurricular activities were conducted but were organized only for special events. This finding was consistent with the related study of nursing students, which found that no subjects in the nursing curriculum contained tobacco control entirely^29^. However, during four years of study in public health education, institutions should be able to develop students as members of health teams who have gained health consciousness by serving as non-smoking role models for the general public. Even with current constraints on the public health education system regarding a core subject entirely committed to tobacco control, this might be accomplished through various methods. For example, the content could be included in core or elective curriculum subjects. It could be developed as an e-learning course that students must enroll in as a professional soft skills course before graduating. The e-learning modules have shown much better results regarding knowledge of tobacco issues for professional students, particularly medical students^30,31^. It could also be achieved by setting the condition that ‘tobacco control’ is a required topic for a field training program. For example, institutions could conduct a case study about assessing quitting smoking readiness, offer cessation services according to the process of change, create a smoke-free environment in the community, organize a learning opportunity among related sectors in the community and provide feedback. These activities will develop students’ skills in searching, systematic thinking, and applying knowledge to action to solve problems^32,33^.

Moreover, the PRECEDE-PROCEED Model^19^ in this study showed that all three factors significantly explained the students’ e-cigarette use. One of the predisposing factors found to be significantly associated with e-cigarette use was attitude. Subjects with a neutral attitude toward e-cigarette use were 2.2 times more likely to use e-cigarettes than those with a negative attitude toward e-cigarettes because individuals’ attitudes influenced their desire to engage in target behaviors^34^. Regarding the role model concept, subjects perceiving that public health professionals should not serve as non-smoking role models used an e-cigarette 2.3 times more than those perceiving public health professionals should serve as non-smoking role models. It reflected that when the teaching and learning process can develop learners’ perceptions that public health professionals should serve as models for good health among non-smokers, it would be beneficial for them to be non-smokers. Further, other people could be engaged due to their motivation and gain the expectation of that health outcome as role models^35^.

Concerning enabling factors, subjects who had tried any tobacco products had used e-cigarettes 40.7 times more than those who had never tried. It could be because e-cigarettes have a distinct flavor and odor and an attractive container package design more than traditional cigarettes. For the reinforcing factor, close friends’ smoking influenced subsequent smoking 3.2 times due to being stimulated or persuaded by peers, leading to their continued smoking. This finding was consistent with related studies showing that those having friends who smoked used cigarettes more than 5.6 times (95% CI: 3.6–8.8)^36^ and e-cigarettes more than 2.6 times (95% CI: 1.4–5.1) than those not having peers who smoked^37^. These findings suggest that adolescents need to be a part of a group in their environment. They prefer to spend a significant amount of time with their friends daily, leading to imitation, conformance, or compliance with their peers to be accepted and reduce conflicts in their group^38^.

### Strengths and limitations

This study provided Thai baseline data on e-cigarette use among public health students. Their baseline data will assist public health networks in following up on e-cigarette use and designing appropriate supportive teaching and learning activities within the curricula and extracurricular activities. Furthermore, an online questionnaire was designed to be brief and precise, motivate subjects, and reduce boredom by providing information. The study encountered limitations. First, the primary dataset from a cross-sectional online survey may limit the ability to detect causal relationships compared with a structural causal model. The second limitation is the self-reported questionnaire. Even though the questionnaires were reliable, youth smoking remains a sensitive issue due to Thai culture, which still values non-smoking youths.

## CONCLUSIONS

The current e-cigarette users among third-year public health students were associated with five significant factors: sex, attitude, being a role model, having tried tobacco products, and the number of close friends who smoked. These factors can cause e-cigarette smoking initiation and continuation. Thus, to prevent public health profession students from becoming new smokers, developing students through various teaching and learning activities would be significant, such as: develop students’ awareness of serving as non-smoking role models; promote a smoke-free environment both in the institutes and the surrounding community; and develop a student database for monitoring smoking situations, and assist them in successfully quitting smoking.

## Data Availability

The data supporting this research are available from the authors on reasonable request.
