# Supplementary material for: Use of e-cigarettes among public health students in Thailand: Embedded mixed-methods design
Source: Tob Induc Dis. 2022 Sep 7;20:78. doi: 10.18332/tid/152256 (PMC9449824; doi:10.18332/tid/152256)
Supplement: Supplementary file 1 [file TID-20-78-s1.pdf]

Online survey of health profession for tobacco control, 2021

Final version in hard copy format as of October 6, 2020

Section 1: Tobacco use

1. Have you ever tried any smoked tobacco products, even 1 – 2 puffs in their lifetime? (For example, manufactured cigarette, e-cigarette, hand-rolled cigarette, and others)

   1) NO 2) Yes

2. During the past 30 days, have you smoke any tobacco products as the follow's items? (answer every item)

2.1 Manufactured cigarette	1) No 2) Yes
2.2 E-cigarette	1) No 2) Yes
2.3 Hand-rolled cigarette	1) No 2) Yes
2.4 Others such as baraku, pipe, and cigar	1) No 2) Yes

3. During the past 30 days, how many days do you smoke the following products (answer every item)

3.1 Manufactured cigarette	Possible code ranges from 0 for none, and 1 – 30 days)
3.2 E -cigarette	Possible code ranges from 0 for none, and 1 – 30 days)
3.3 Hand-rolled cigarette	Possible code ranges from 0 for none, and 1 – 30 days)
3.4 Others such as baraku, pipe, and cigar	Possible code ranges from 0 for none, and 1 – 30 days)

4. During the past 30 days, how many of the following products do you smoke each day? (answer every item)

**4.1** Manufactured cigarette	Possible code ranges from 0 for none, and 1 – 40 sticks/ day
**4.2** E -cigarette	Possible code ranges from 0 for none, and 1 – 20 round/ day<br>If respondents report 1 round and over per day, go to 4.2.1<br>**4.2.1** each round, How many time of vaping?
**4.3** Hand-rolled cigarette	Possible code ranges from 0 for none, and 1 – 40 sticks/ day
**4.4** Others such as baraku, pipe, and cigar	Possible code ranges from 0 for none, and 1 – 20 times/ day

5. During the past 30 days, where did you obtain tobacco items the most?
   1) I have never smoked in lifetime
   2) Convenient shop
   3) Grocery
   4) Online shop
   5) Others such as buying from agency, buying from friends, or asking friends to buy

Section 2: Secondhand smoke

6. Do you have a close friend in the institute smoked tobacco products such as manufactured cigarette, e-cigarette, hand-rolled cigarette, and others?

   ☐☐ [Possible code ranks from 0 (no or don't know) and 1 to xx person]

7. During the past 7 days, how many days did someone smoking nearby you in the institute?

   ☐ (Possible code rank from 0 – 7 days)

8. Do you know the law that prohibits smoking all tobacco products including e-cigarette in the institute?

   1) No 2) Yes

9. Did you notice a no smoking sign outside the building of the institute?

   1) No 2) Yes

10. Did you notice a no smoking sign inside the building of the institute?

   1) No 2) Yes

Section 3: Cessation

11. How soon after you wake up do you usually use smoked tobacco products for the first time?
   1) I have never smoked in lifetime
   2) I used to smoke, but now I don't smoke
   3) less than 10 minutes
   4) 10 – 30 minutes
   5) 31 – 60 minutes
   6) more than 60 minutes

12. Did you think about quitting smoking?
   1) I have never smoked in lifetime
   2) I used to smoke, but now I don't smoke
   3) I don't thinking about quitting smoking
   4) I want to quit, but I can't commit to a certain day.
   5) I want to quit within the next 6 months
   6) I want to quit within the next 30 days

13. During the past 12 months, have you tried to quit smoking?
   1) I have never smoked in lifetime
   2) I did not smoke during the past 12 months
   3) I have never tried to quit smoking during the past 12 months
   4) I have ever tried to quit smoking during the past 12 months

   → 13.1 specific: ☐☐ times (possible code rank from 1 -12)

14. Have you ever had received advice or assistance to not smoke or quit smoking?
   1) I have never smoked in lifetime
   2) No
   3) Yes

Section 4 **Teaching and learning about tobacco control**

15. In your opinion, should health professionals serve as nonsmoking role models for clients and the general public?
   1) No   2) Yes

16. In your opinion, should health professional advise patients or clients who use tobacco products to quit smoking?
   1) No   2) Yes

17. During studies at the institution, have you ever attended a class on the following topics related to tobacco control? (Answer every items)
   17.1 Tobacco's hazards   1) Never   2) Ever
   17.2 Technique of quitting cigarette smoking   1) Never   2) Ever
   17.3 Drugs and products used for quit smoking   1) Never   2) Ever
   17.4 marketing's strategies of the tobacco industry   1) Never   2) Ever

18. During the past 12 months, have you noticed an anti-smoking campaign in the institute?
   1) Never   2) Ever

19. During the past 12 months, have you ever taken part in an anti-smoking campaign at the institute?
   1) Never   2) Ever

Section 5 **Attitude towards e-cigarettes use**

items	question	1 Strongly disagree	2 disagree	3 neutral	4 agree	5 Strongly agree
20.	E-cigarettes are safer than traditional cigarettes					
21.	E-cigarette smoking shows modernity and being in the new generation					
22.	E-cigarette use is not illegal					
23.	E-cigarettes are not addictive					

items	question	1<br>Strongly disagree	2<br>disagree	3<br>neutral	4<br>agree	5<br>Strongly agree
24.	E-cigarettes can reduce the chances of developing health problems such as emphysema, cancer, and coronary artery disease					
25.	E-cigarettes can help to quit traditional cigarettes					
26.	Use of e-cigarettes represent modernity and a new generation					
27.	The modern image of e-cigarette arouses curiosity to try					
28.	Use of e-cigarettes makes it easy to get along with friends					

Section **6** Background information about yourself

29. What year you are in university?

A. graduated
B. Year 1
C. Year 2
D. Year 3
E. Year 4
F. Year 5
G. Year 6
H. Year 7

30. What is your health profession's field of study? ท่านเป็นนิสิตนักศึกษา / บุคลากรวิชาชีพสุขภาพสาขาใด

A. Medicine
B. Nursing
C. Pharmacy
D. Dentist
E. Physical therapy
F. Medical technologist
G. Public Health

31. What is your sex?

1) Male 2) Female

32. How old are you? ☐☐ Years old

33. What is your institution name? ....

© 2022 Benjakul S. et al.
